# Ecological drivers of plant genetic diversity at the southern edge of geographical distributions: Forestal vines in a temperate region

**DOI:** 10.1590/1678-4685-GMB-2017-0031

**Published:** 2018

**Authors:** Michel J.F. Barros, José Alexandre F. Diniz-Filho, Loreta B. Freitas

**Affiliations:** 1 Department of Genetics, Universidade Federal do Rio Grande do Sul, Porto Alegre, RS, Brazil; 2 Department of Ecology, Universidade Federal de Goiás, Campus II, Goiânia, GO, Brazil

**Keywords:** Distribution edges, genetic diversity, Passiflora, niche conservatism, environmental drivers

## Abstract

The Tropical Niche Conservatism hypothesis is one of the most relevant theories to explain why tropical diversity is high, although the mechanisms underlying this hypothesis require further clarification. A possible research avenue to address the underlying mechanisms includes determining population-level processes associated with such a hypothesis, in particular by trying to identify how adaptation may occur in extreme niche conditions at the edges of species ranges. However, the determinants of molecular diversity at the edges of geographical distributions of tropical taxa are still poorly known. Here we assessed which environmental variables determine diversity in nuclear and plastid genetic markers for populations of four *Passiflora* species in the southern limit of their geographical distributions. Climatic factors can drive genetic diversity, and their importance varies according to the marker. The primary predictors are variables representing higher temperatures during cold periods of the year and higher precipitation during dry periods. We concluded that, although these species are present in colder areas at the edge of their range, Tropical Niche Conservatism acts as a restraining force on genetic diversity in southern populations of *Passiflora*.

## Introduction

Understanding the limits of geographical distribution of species and how these limits are determined is of special interest to answer several questions in evolution and population genetics (Angert, 2009). Distribution limits vary among the different levels of evolutionary organization (among species in clades, among clades) and across the geography per se (Sheth and Angert, 2014) and several hypotheses have been proposed to clarify these differences. Many studies have associated the limits of geographical range to limiting environmental variables (Angert, 2009), and range limits for many species are at least partially imposed by climate gradients that will shift directionally under climate change (Parmesan, 2006; Klimeš and Doležal, 2010).

The Rapoport’s rule states that species can have narrower tolerances in more stable climates, leading to smaller ranges and allowing coexistence of more species (Sizling *et al.*, 2009), and the pattern of average range size in a clade would decrease from temperate to tropical areas (Sheth and Angert, 2014). Conversely, tropical species could have more specialized habitat requirements and narrower tolerances than temperate taxa (Stevens, 1989). Thus, according to Angert (2009), species distribution can be viewed as a spatial manifestation of the niche, where the geographical range represents a mapping of fitness as a function of abiotic and biotic environment on landscape.

The tropical niche conservatism (TNC) hypothesis has frequently been used to explain why tropical ecosystems are biologically more diverse (Wiens and Donoghue, 2004; Diniz-Filho *et al.*, 2007; Oliveira-Filho *et al.*, 2013; Hawkins *et al.*, 2014). Among other statements, this model suggests that dispersal into temperate regions has been limited because adaptation to colder temperatures appears to be uncommon (Smith *et al.*, 2012). Yet, despite its importance, the mechanisms underlying TNC are still poorly understood, in large part because many studies address macroecological data only.

Plant families that originated in the South generally exhibit higher TNC values and include many lineages restricted to the Neotropics, whereas several northern-origin groups occur both in the Neotropics and in Nearctic regions (Smith *et al.*, 2012). Accordingly, the TNC model states that few tropical groups expanded and adapted to temperate environments (Wiens and Donoghue, 2004). Therefore, the influence of factors such as demographic stochasticity might participate in shaping the distribution of diversity at the margins of species’ ranges (Hampe and Petit, 2005).

*Passiflora* L. is the largest genus in Passifloraceae and encompasses ca. 560 wild species distributed primarily in the Neotropical region (Ulmer and MacDougal, 2004). Only approximately 5% of these species are distributed outside of tropical Central and South America (Abrahamczyk *et al.*, 2014). The most recent molecular phylogeny has organized *Passiflora* species into four subgenera that are fully supported by morphological traits (Muschner *et al.*, 2012). Two of them present several important genetic differences, such as genome size (Yotoko *et al.*, 2011) and organellar inheritance (Muschner *et al.*, 2006). The *Passiflora* species have been used to explain the origin of existing forests in the temperate zone in southern Brazil (Rambo, 1961), and they are related to the biogeography of this region (Moreira *et al.*, 2011).

The Brazilian state of Rio Grande do Sul (RS) is the extreme southern limit of 16 species of *Passiflora* (Cervi, 2006; Mondin *et al.*, 2011), and here we selected these to investigate environmental variables that determine variability in nuclear and plastid genetic markers for populations at the southern limit of *Passiflora* species’ geographical distribution and associate them to different physiographical regions.

## Materials and Methods

### Studied areas

We defined our study region to assess the differentiation of physiographic regions from the north of Rio Grande do Sul, Brazil (Figure S1) as described in Fortes (1959). We focused on the region called “Portal de Torres’, a transitional area for diversity in the eastern tropical forests of Brazil (Pinheiro *et al.*, 2011) and, to the west, the Upper Paran and Uruguay Rivers. These are the areas in which *Passiflora* species occur in the extreme south of Brazil. We also sampled information from the east of Rio Grande do Sul, representing a more humid area at the southern limit of the distribution of many species within the *Passiflora* genus (Mondin *et al.*, 2011; Moreira *et al.*, 2011).

### Data sampling and handling

We extracted environmental data and biogeographical information for the entire region where *Passiflora* species are found, from the Portal de Torres to the west, and environmental and genetic information from the east representing the southern limit for four *Passiflora* species (*P. caerulea* L. and *P. tenuifila* Killip, representing the *Passiflora* subgenus, and *P. misera* Kunth and *P. capsularis* L., representing the *Decaloba* subgenus).

For the environmental analyses we obtained information on climate and altitude variables in occurrence areas of *Passiflora* to assess the degree of variance in different environmental factors in the studied region and search for multivariate environmental differentiation among major areas. We used altitude and bioclimatic variables obtained from WORDCLIM (http://www.worldclim.org/). More specifically, 19 variables representing distinct properties of climate such as temperature- and precipitation-specific niche characteristics were included in this study. These variables are widely used for modelling niches of species and other taxa because they represent specific environmental factors that can influence the geographical distribution of taxa on large and small scales. We used layers with a resolution of 2.5 arc minutes (~4.5 km) and extracted average values per grid cell. We transformed point data into grid files by implementing the re-sampling circular neighborhood technique (as suggested by Zonneveld *et al.*, 2012), which we applied in Diva-GIS 7.5.0 (Hijmans *et al.*, 2002).

To access genetic variability, we selected two relevant markers for our analyses: the nuclear ribosomal DNA internal transcribed spacers (ITS) and the plastid intergenic spacer *trnH*-*psbA*. The ITS sequences present moderate to high intraspecific genetic variability in *Passiflora* (Mäder *et al.*, 2010; Giudicelli *et al.*, 2015), and *trnH*-*psbA* is one of the most variable regions in Angiosperm genomes (Storchov and Olson, 2007). DNA sequences were obtained using primers and protocols described by Lorenz-Lemke *et al.* (2005). We obtained sequences from 31 individuals from *P. caerulea,* 30 from *P. tenuifila,* 18 from *P. misera,* and 26 from *P. capsularis*. Geographical coordinates obtained from previous fieldwork represented all individuals (Supplementary Material Table S1). To avoid incorrect *a priori* definitions of populations, we included molecular richness grouping data from all four species, defined according to their occurrence in distinct spatial units, so that these data included all sequence types per marker (see also Miraldo *et al.*, 2016). Specifically, we mapped the richness of sequences in the grid cells and used the richness scores as response variables representing the genetic structure of markers. As the same individuals were used to represent both genetic markers, we developed two grids layers, formed by 134 cells each, at 0.3 degrees of resolution. The cells obtained were the same ones used for the extraction of values of environmental factors, which we applied for the subsequent analyses, including modelling of genetic diversity.

### Data analyses

We used principal component analyses (PCA) based on the values of climate and altitude variables in each grid cell with occurrence of *Passiflora* to assess the degree of variance in different environmental factors in the studied region and search for multivariate environmental differentiation among major areas. The four resulting PCs were used to establish K-means clusters for two and three groups, consecutively. Biogeographical cluster analyses were implemented based on a presence/absence matrix of the species of *Passiflora* following occurrences reported by Mondin *et al.* (2011) in the 11 physiographic regions proposed by Fortes (1959). To accomplish this, we used UPGMA (Hammer *et al.*, 2001) based on the Euclidian distance among physiographic regions and bootstrapped with 1000 randomizations. Subsequently, to provide additional robustness to the presence/absence matrix, we integrated this dataset with a matrix for the genus *Mikania* (Ritter and Waechter, 2004), which also takes into account the physiographic regions defined by Fortes (1959).

The sequences of both genetic markers were automatically aligned using Muscle as implemented in MEGA 6.0 (Tamura *et al.*, 2013). Molecular diversity indices were calculated in DnaSP 5.0 (Librado and Rozas, 2009) for the four species. Sequence richness was used for estimating genetic diversity within species and for spatial analysis because it has already been shown as strongly correlated to species definition as well as environmental predictors (Papadopoulou *et al.*, 2011). Nucleotide diversity was also estimated, but it was not high enough to be used in correlation analyses.

We applied three spatial regression methods (see Diniz-Filho *et al.*, 2009) to search for regional and local predictors of diversity: ordinary least squares (OLS), simultaneous auto-regressive (SAR) models, and geographically weighted regressions (GWR), all implemented in the software SAM 4.0 (Rangel *et al.*, 2010) using the grid cells as geographical units. For OLS and SAR, we normalized the distributions of the variables’ values to homogenize the variances (*i.e.*, create homoscedasticity) by applying average environmental values per diversity class obtained in the diversity grids (*e.g.*, using all grid cells represented by one sequence type, we computed the average values of the environmental variables; we did this for each diversity class). We used Shapiro-Wilk, Jarque-Bera, Chi-square, and Anderson-Darling tests to assess the normality of the distributions, as implemented in PAST 2.17 (Hammer *et al.*, 2001).

To select models and to identify the most relevant environmental predictors from autoregressive models, we used the Akaike information criterion (AIC), which takes into account both predictive power and complexity (Rangel *et al.*, 2010). In addition to the total coefficients obtained by OLS and SAR, we used GWR to obtain local and spatially explicit model parameters for each grid cell, based on ITS and *trnH*-*psbA* markers. We grouped together all environmental predictors in SAR models selected by AIC for both genetic markers. Next, the inclusion of additional variables for GWR was assessed regarding its power to predict the diversity, minimizing the residuals, and increasing the model effectiveness. In GWR, according to a predefined function, all data points within a given bandwidth can be weighted according to their spatial distance from a focal point. The distance decay of the weight adopted was a Gaussian function with a fixed bandwidth established via AIC optimization. We assessed the residuals of the GWR models for each grid cell and at distinct distance classes for both models representing the molecular markers.

## Results

### Clustering biogeographical and habitat areas

**Figure 1:**
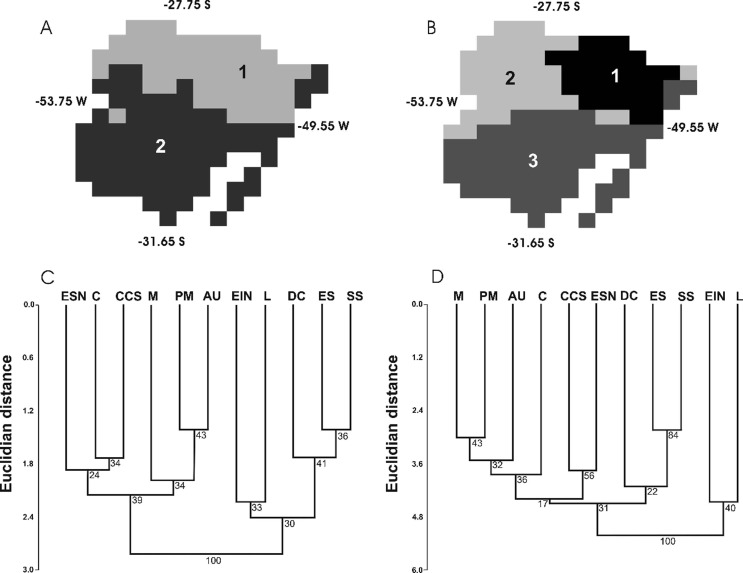
Geographical clusters derived from the four most relevant principal components under K = 2 (A) and K = 3 (B). Cluster analyses for vegetation groups based on a paired group algorithm using Euclidian distances bootstrapped by 1000 randomizations based on either all *Passiflora* species occurrence (C) or *Passiflora* and *Mikania*, incorporating the distance matrix from Ritter and Waechter (2004) (D). The physiographical regions according to Fortes (1959): Litoral (L), Depresso Central (DC), Encosta Inferior do Nordeste (EIN), Campos de Cima da Serra (CCS), Encosta Superior do Nordeste (ESN), Misses (M), Planalto Médio (PM), Alto Uruguai (AU), Campanha (C), Serra do Sudeste (SS), and Encosta do Sudeste (ES).

Individuals of *Passiflora* were found in three physiographical regions according to the classification by Fortes (1959) for southern Brazil: Encosta Inferior do Nordeste (EIN), Encosta Superior do Nordeste (ESN) and Litoral (L), and these were included in the genetic analyses (Table S1). A distinction between eastern and western physiographical areas proposed by Fortes (1959) was confirmed in geographical analyses (Figure 1A). A similar separation did not appear in the K = 2 environmental groups (Figure 1A), but became more evident in the K = 3 (Figure 1B). The cluster analyses of phytogeographical regions, based on the presence/absence matrix of species of *Passiflora* (Figure 1C) and *Passiflora* + *Mikania* (Figure 1D), validated the separation between the east and west that the biogeographical groups largely defined (full bootstrap; Figure 1C,D). These groups primarily consisted of separate western and eastern portions of northern forests in Rio Grande do Sul, which are part of the Atlantic Forest domain.

The four principal components determined by PCA based on environmental data, which were also used for clustering analyses (K-means), explained ~92% of the total environmental variance (Table 1). PC 1 alone accounted for ~56% of the total variance and was principally formed by altitude and the following bioclimatic variables: mean temperature of the warmest quarter, annual precipitation and annual mean temperature. Therefore, most of the differentiation among niches identified in the geographical clustering analyses using altitude and bioclimatic variables derived from these specific variables because their variances and explanatory power were higher (Table 1). The eastern areas are more heterogeneous and most likely present more diverse niches; this becomes more evident with K = 3 than with K = 2 (Figure 1A,B). Additionally, there is a differentiation between eastern and western niches. Following this environmental configuration, a group representing the Pampas and Coastal ecosystems appears as a separate unit (Figure 1B; group 3) and includes most of the modelled genetic diversity. Although it was not our objective here, we highlight that more variance can be found within this last group, which separates the resting plus rain forest physiognomies in the littoral zone from the Pampas grassland.

**Table 1:** The most relevant niche contributors identified by principal component analysis. The values of environmental variables were extracted for each grid cell. Here we present the four principal components, which explained most of the total environmental variance (91.66%). The variance (V) and eigenvalues (E) are shown for each PC, while we presented the loadings (L) for each identified contributor.

	V (%)	E	Environmental contributors	L
PC1	55.56	11.11	Altitude	0.28
			Mean Temperature of Warmest Quarter	-0.27
			Annual Precipitation	0.27
			Annual Mean Temperature	-0.26
PC2	16.47	3.29	Mean Temperature of Wettest Quarter	0.44
			Mean Diurnal Range	0.36
			Precipitation of Coldest Quarter	-0.32
			Mean Temperature of Driest Quarter	-0.30
PC3	12.7	2.55	Precipitation Seasonality	-0.38
			Temperature Annual Range	0.35
			Mean Diurnal Range	0.33
			Precipitation of Driest Quarter	0.31
PC4	6.87	1.37	Temperature Annual Range	0.44
			Mean Temperature of Wettest Quarter	-0.34
			Precipitation Seasonality	0.33
			Min Temperature of Coldest Month	-0.32

### Diversity and niche predictors

Total genetic diversity is presented in Table 2. Re-sampling sequence richness was useful to the study of both markers. Sequence richness for the ITS grid was represented by values ranging from 1 to 23. The *trnH*-*psbA* grid presented values of richness ranging from 1 to 24. A clear difference in coefficients was observed in a comparison of OLS and SAR models (Table 3). Inflation in OLS coefficients was observed for two predictors of nuclear diversity, and underestimates were observed for the prediction of plastid diversity. To select the most relevant predictors, we looked at the models that presented the lowest AIC values in SAR. These models indicated that diversity in the ITS marker was highly correlated with temperature predictors, while precipitation variables better explained the occurrence of plastid diversity (Table 3). These niche determinants reflected the adaptation of *Passiflora* to tropical warm and humid climates. The diversity variables were strongly correlated with the amount of higher temperatures and the elevated occurrence of precipitation in harsh annual periods.

**Table 2:** Characteristics of DNA sequences and summary of genetic diversity of four *Passiflora* species.

	Genetic Marker	*P. caerulea*	*P. tenuifila*	*P. misera*	*P. capsularis*
Sequence length (bp)	ITS	385	478	607	634
	*trnH-psbA*	325	319	290	362
Sequence types (n)	ITS	12	11	5	5
	*trnH-psbA*	4	8	3	3
Genetic diversity^*^	ITS	0.847 (0.046)	0.916 (0.041)	0.713 (0.077)	0.762 (0.058)
	*trnH-psbA*	0.440 (0.009)	0.484 (0.113)	0.205 (0.119)	0.324 (0.207)

* Standard deviation in parenthesis

**Table 3:** Selected values of correlation coefficients. The best SAR models were selected based on minimizing AIC values.

Genetic marker	Niche variables	SAR	OLS	AIC	p
ITS	Mean Diurnal Range (BIO2)	0.458	0.513	152.78	0.016
	Min Temperature of Coldest Month (BIO6)	0.562	0.418	149.58	0.054
	Temperature Annual Range (BIO7)	0.334	0.408	155.33	0.061
*trnH-psbA*	Precipitation of Driest Quarter (BIO17)	0.905	0.522	125.68	0.012
	Precipitation of Driest Month (BIO14)	0.999	0.453	10.626	0.032
	Annual Precipitation (BIO12)	0.993	0.374	67.534	0.079

**Figure 2:**
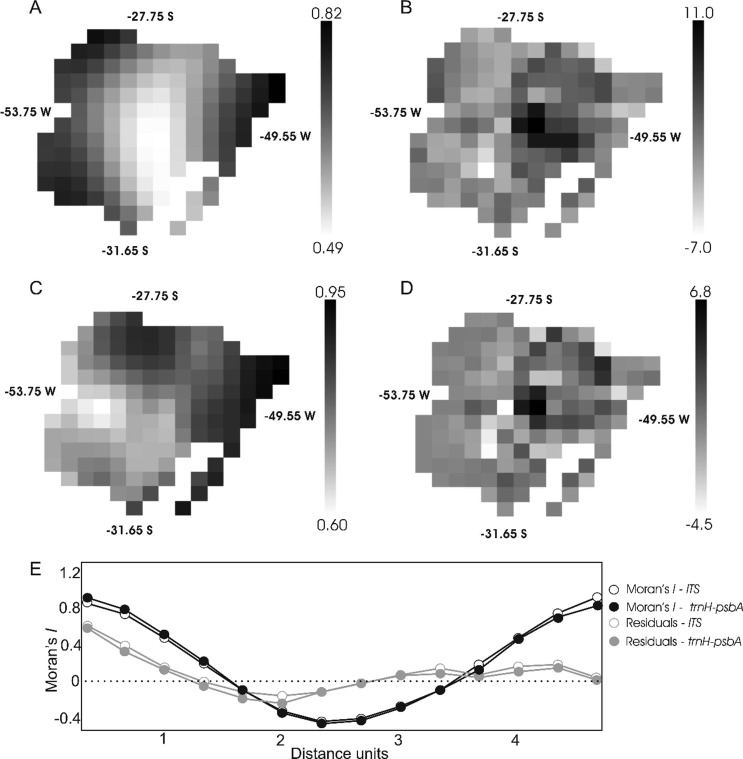
Geographically weighted regression. The values of genetic diversity were regionally regressed against the most relevant bioclimatic predictors identified in SAR models (Table 3) in addition to altitude to minimize residuals. The ITS marker exhibited higher local *R* ^2^ values in eastern and western areas (A), and the highest values of residuals were minimized in these areas (B). The local *R* ^2^ for *trnH*-*psbA* are lower in the central area (C), with a pattern of residual values similar to that observed for ITS (D). All analyses were significant (p < 0.001). The Moran’s *I* correlogram (E) shows that these similarities in correlation and residuals are both also found in distinct distance classes.

GWR analyses revealed similar patterns of local *R* ^2^ for both DNA markers, and the inclusion of temperature and precipitation variables identified in SAR (Table 3), plus altitude, was the combination that minimized model residuals (Figure 2). However, the coefficients estimated based on ITS data predicted diversity principally in the northwest, northeast coastal, and southwestern areas of species distributions (Figure 2A,B), whereas higher predictions for plastid markers were obtained in the eastern, coastal, and northwest regions (Figure 2C,D). The autocorrelation of residuals was minimized for distant regions and when the variable altitude was included. However, even with the inclusion of altitude, it remained high in the first distance class (Figure 2E). Anyway, most of the grid cells with high degrees of residual variance were outside the predicted hotspots of diversity (Figure 2B,D).

## Discussion

### Dealing with spatial autocorrelation in genetic diversity

Spatial patterns can be better interpreted when autocorrelation in trait values is considered. Legendre (1993; see also Dormann *et al.*, 2007) postulates that spatial analysts may interpret spatial autocorrelation as an opportunity or a challenge. In fact, it is an important property of biological diversity that can result from evolutionary and ecological phenomena. Therefore, our findings were successful in combining simultaneous autoregressive (SAR) models to identify predictors and geographically weighted regressions (GWR) analysis of local correlations to search for ecological drivers of genetic variability at the scale of grid cells.

Although the studied areas belong to a region with a temperate climate, genetic patterns found based on molecular diversity of *Passiflora* are more closely related to representative variables of environmental conditions from tropical zones. This suggests that, despite reaching temperate zones in the southern limit of the genus’ geographical distribution, these species only occur in suitable (tropical-like) conditions and do not present patterns driven by the temperate climate itself. Instead, they may be constrained by the typical temperate climate (broadly including subtropical climates). The use of AIC allowed us to select the minimum adequate autoregressive models and suggested that increases in the values of temperature variables better predict diversity in the nuclear marker ITS, while the amount of precipitation is positively associated with plastid variability (Table 3). All these findings suggest a dependency of tropical climate for diversification, demonstrating the possibility that TNC plays an important role to genetic variability of *Passiflora* in the edge of its distribution. However, more species and markers within the genus should be tested in the future using methods that incorporate the effect of spatial autocorrelation to confirm this proposition.

### Origin and differentiation of diversity areas in southern forests

The division into western and eastern forests, previously suggested by Ritter and Waechter (2004), was confirmed here for the region studied based on species occurrence and clusters of multivariate components, although the eastern areas are more heterogeneous and present a higher number of niches (Figure 1A,B). The eastern forests studied here lie in the southernmost area and are more propitious to the occurrence of *Passiflora* species. It is a region with high *Passiflora* species richness (L.B. Freitas, unpublished data; Mondin *et al.*, 2011), despite the fact that cold periods are more common in this region than in forests located farther north.

Migration from typically tropical areas to areas with colder climatic conditions is not common and contributes to the accumulation of species in tropical regions (Wiens and Donoghue, 2004; Diniz-Filho *et al.*, 2007). It is the environmental suitability for taxa with tropical characteristics that forms the conditions that allow the occupation of these areas. This is also true for the model of diversity structure studied here. The genetic diversity found in *Passiflora* species is also most likely derived from historical migrations through the `Portal de Torres’ as well as from the diversification of molecular types that are autochthonous and resilient in the South and remained conserved in tropical-like niches. In fact, both niche suitability and migration appear to be the dominant causes of spatial patterns of species turnover and diversity of genetic markers in the region studied.

The suitability of this region for forest taxa is most likely a result of landscape patterns. The northwestern and eastern forest areas occupy the region surrounding the Brazilian plateau and are apparently dependent on the warm and moist characteristics of these large areas. Additionally, altitudes gradually increase toward the north and northwestern areas of the Rio Grande do Sul state, reaching approximately 900 meters above sea level in the north. In fact, altitude was the variable with the greatest contribution according to PCA (Table 1). The forests in the eastern sites (the area sampled for modelling genetic diversity) occur in lowlands and areas with lower altitudes in the slopes of the ÒSerra GeralÓ. There are species that occur in both eastern and western forests and other species that occur in only one of these areas (Moreira *et al.*, 2011), most likely reflecting environmental and historical influences. In addition to the longitudinal diversity gradient, the variations with latitude are directly related to resistance to cold conditions.

The species *P. caerulea* is known for its hardiness in the winter and resistance to temperate climates (Yockteng *et al.*, 2011). Recently, Sheth and Angert (2014) demonstrated that species with high thermal tolerance also present high genetic diversity. In fact, the populations of *P. caerulea,* as well as those from *P. tenuifila,* exhibited higher diversity indices for both markers than *P. misera* or *P. capsularis*, indeed the diversity indices were similar to those obtained for *P. actinia* Hook. (subgenus *Passiflora*; Teixeira *et al.*, 2016) or *P. contracta* Vitta (subgenus *Deidamioides*; Cazé *et al.*, 2016) from the Atlantic Forest. In fact, *P. caerulea* is one of the species reaching the southernmost areas of the distribution of the genus (Moreira *et al.*, 2011). Thus, the transition from tropical to temperate areas might be highly propitious for molecular diversification in *Passiflora* species, and this pattern may be amplified when breaks in the distribution are taken into account (Moreira *et al.*, 2011).

Stochasticity was cited as a relevant factor in determining distribution range limits (Hampe and Petit, 2005) and our results complementarily indicate that conservatism is also relevant for genetic diversity in the spatial limits of niches. In fact, vertebrate taxa typical of South America have previously been reported to have high TNC rates (Diniz-Filho *et al.*, 2007; Smith *et al.*, 2012). Similar preferences were also verified for several tropical plant taxa (Oliveira-Filho *et al.*, 2013; Hawkins *et al.*, 2014). We also observed that thermal variation may cause population-level stochasticity (Hampe and Petit, 2005), and breaks could promote isolation of several *Passiflora* species (Moreira *et al.*, 2011). In addition to these factors, we verified a high correlation with tropical climate, as shown here and in line with TNC, these might be the major determinants of the structure and genetic diversity in *Passiflora* populations at the southern limit of the species’ distribution.

## Supplementary material

The following online material is available for this article:

Table S1 - Sampling information.

Figure S1 - State of Rio Grande do Sul in the context of South America.
