# Supplementary material for: Ecological drivers of plant genetic diversity at the southern edge of geographical distributions: Forestal vines in a temperate region
Source: Genet Mol Biol. 2018;41(1 Suppl 1):318–26. doi: 10.1590/1678-4685-GMB-2017-0031 (PMC5913715; doi:10.1590/1678-4685-GMB-2017-0031)
Supplement: Supplementary file 1 [file 1415-4757-GMB-41-01-2017-0031-s002.pdf]

Supplementary Material to “Ecological drivers of plant genetic diversity at the southern edge of geographical distributions: Forestal vines in a temperate region”

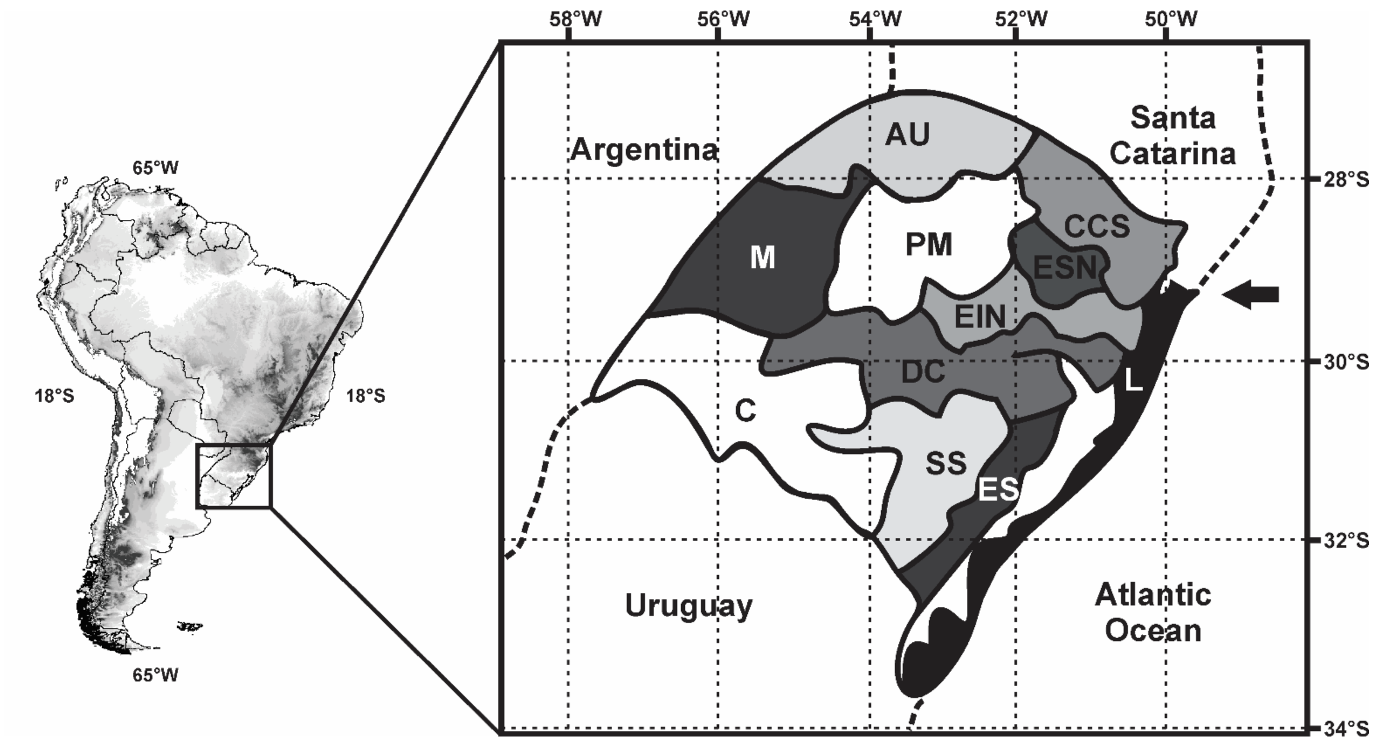

Figure S1 - The phytogeographical regions of the state Rio Grande do Sul in Brazil,as proposed by Fortes (1959) and used in this work: Litoral (L), Depressão Central (DC), Encosta Inferior do Nordeste (EIN), Campos de Cima da Serra (CCS), Encosta Superior do Nordeste (ESN), Missões (M), Planalto Médio (PM), Alto Uruguai (AU), Campanha (C), Serra do Sudeste (SS), and Encosta do Sudeste (ES). The arrow indicates the position of the Portal de Torres, an area of high biogeographical interest because of tropical species migration to the temperate zone, thus representing a transitional area (Pinheiro *et al.*, 2011).
