# Supplementary material for: Ecological drivers of plant genetic diversity at the southern edge of geographical distributions: Forestal vines in a temperate region
Source: Genet Mol Biol. 2018;41(1 Suppl 1):318–26. doi: 10.1590/1678-4685-GMB-2017-0031 (PMC5913715; doi:10.1590/1678-4685-GMB-2017-0031)
Supplement: Supplementary file 2 [file 1415-4757-GMB-41-01-2017-0031-s001.pdf]

Supplementary Material to "Ecological drivers of plant genetic diversity at the southern edge of geographical distributions: Forestal vines in a temperate region"

Table S1 - Sampling information.

Species	Individual	Longitude	Latitude	ITS*	*trnH-psbA**
P. caerulea	C12	-51.1172	-30.0779	KP223326	KP223411
P. caerulea	C13	-51.1172	-30.0779	KP223327	KP223412
P. caerulea	C14	-51.1172	-30.0779	KP223328	KP223413
P. caerulea	C15	-51.323	-30.1665	KP223329	KP223414
P. caerulea	C17	-50.6106	-29.4383	KP223330	KP223415
P. caerulea	C19	-51.1875	-29.0261	KP223331	KP223416
P. caerulea	C20	-51.1875	-29.0261	KP223332	KP223417
P. caerulea	C23	-50.6287	-29.4558	KP223333	KP223418
P. caerulea	C30	-50.8678	-29.3904	KP223334	KP223419
P. caerulea	C31	-51.1136	-29.3741	KP223335	KP223420
P. caerulea	C32	-50.8119	-29.356	KP223336	KP223421
P. caerulea	C36	-50.8813	-30.1348	KP223337	KP223422
P. caerulea	C45	-51.1875	-29.0261	KP223338	KP223423
P. caerulea	C48	-51.1792	-29.1629	KP223339	KP223424
P. caerulea	C49	-51.3098	-29.0275	KP223340	KP223425
P. caerulea	C51	-51.3098	-29.0275	KP223341	KP223426
P. caerulea	C52	-51.3098	-29.0275	KP223342	KP223427
P. caerulea	C61	-50.4663	-29.8041	KP223343	KP223428
P. caerulea	C62	-50.5111	-29.7307	KP223344	KP223429
P. caerulea	C63	-50.5111	-29.7307	KP223345	KP223430
P. caerulea	C64	-50.4663	-29.8041	KP223346	KP223431
P. caerulea	C67	-50.6698	-29.6444	KP223347	KP223432
P. caerulea	C68	-50.4663	-29.8041	KP223348	KP223433
P. caerulea	C70	-51.5797	-30.0694	KP223349	KP223434
P. caerulea	C107	-51.5568	-30.6396	KP223325	KP223435
P. capsularis	CP05	-51.2883	-28.8565	KP223389	KP223436
P. capsularis	CP14	-50.7217	-29.486	KP223390	KP223437
P. capsularis	CP15	-50.6439	-29.4618	KP223391	KP223438

Species	Individual	Longitude	Latitude	ITS*	*trnH-psbA**
P. capsularis	CP18	-50.4708	-29.807	KP223392	KP223439
P. capsularis	CP19	-50.4708	-29.807	KP223393	KP223440
P. capsularis	CP25	-51.0696	-28.9677	KP223394	KP223441
P. capsularis	CP41	-50.4267	-29.5822	KP223395	KP223442
P. capsularis	CP56	-51.0561	-29.5341	KP223396	KP223443
P. capsularis	CP58	-50.9919	-29.4902	KP223397	KP223444
P. capsularis	CP59	-50.9919	-29.4902	KP223398	KP223445
P. capsularis	CP61	-50.9675	-29.6056	KP223399	KP223446
P. capsularis	CP82	-51.0515	-29.5171	KP223400	KP223447
P. capsularis	CP83	-51.0639	-29.5057	KP223401	KP223448
P. capsularis	CP85	-51.0702	-29.5214	KP223402	KP223449
P. capsularis	CP87	-51.0702	-29.5214	KP223403	KP223450
P. capsularis	CP95	-51.1481	-29.5324	KP223404	KP223451
P. capsularis	CP96	-51.1822	-29.4347	KP223405	KP223452
P. capsularis	CP98	-51.1822	-29.4347	KP223406	KP223453
P. capsularis	CP99	-51.1986	-29.4863	KP223407	KP223454
P. capsularis	CP101	-51.3126	-29.6132	KP223408	KP223455
P. capsularis	CP102	-51.3126	-29.6132	KP223409	KP223456
P. capsularis	CP103	-51.3126	-29.6132	KP223410	KP223457
P. misera	M9	-51.8039	-30.8483	KP223350	KP223458
P. misera	M21	-50.8808	-30.1342	KP223351	KP223459
P. misera	M22	-51.0836	-30.2364	KP223352	KP223460
P. misera	M23	-50.1733	-29.4125	KP223353	KP223461
P. misera	M24	-51.4031	-30.2431	KP223354	KP223462
P. misera	M26	-52.9239	-30.27	KP223355	KP223463
P. misera	M27	-51.3072	-30.1325	KP223356	KP223464
P. misera	M34	-51.5792	-30.0689	KP223357	KP223465
P. misera	M35	-51.6161	-30.0847	KP223358	KP223466
P. misera	M36	-51.6161	-30.0847	KP223359	KP223467
P. misera	M37	-52.0069	-30.1364	KP223360	KP223468
P. misera	M38	-52.0069	-30.1364	KP223361	KP223469
P. misera	M39	-52.0581	-30.1461	KP223362	KP223470

Species	Individual	Longitude	Latitude	ITS*	*trnH-psbA**
P. misera	M40	-52.1447	-30.1614	KP223363	KP223471
P. misera	M41	-52.1447	-30.1614	KP223364	KP223472
P. misera	M46	-50.9894	-30.0953	KP223365	KP223473
P. misera	M49	-51.2444	-29.8744	KP223366	KP223474
P. misera	M52	-52.9258	-30.3194	KP223367	KP223475
P. misera	M53	-51.3342	-30.0508	KP223368	KP223476
P. tenuifila	T4	-51.2067	-30.0317	KP223369	KP223477
P. tenuifila	T5	-51.2067	-30.0317	KP223370	KP223478
P. tenuifila	T8	-51.2239	-30.3186	KP223371	KP223479
P. tenuifila	T10	-51.2067	-30.0317	KP223372	KP223480
P. tenuifila	T16	-51.3097	-29.0275	KP223373	KP223481
P. tenuifila	T17	-51.1875	-29.0261	KP223374	KP223482
P. tenuifila	T18	-51.4878	-30.7733	KP223375	KP223483
P. tenuifila	T19	-51.4878	-30.7733	KP223376	KP223484
P. tenuifila	T20	-51.3086	-30.0578	KP223377	KP223485
P. tenuifila	T21	-51.3086	-30.0578	KP223378	KP223486
P. tenuifila	T22	-51.3086	-30.0578	KP223379	KP223487
P. tenuifila	T26	-50.6844	-29.8897	KP223380	KP223488
P. tenuifila	T27	-51.1911	-29.9353	KP223381	KP223489
P. tenuifila	T28	-52.61	-28.5928	KP223382	KP223490
P. tenuifila	T31	-52.61	-28.5928	KP223383	KP223491
P. tenuifila	T36	-52.61	-28.5928	KP223384	KP223492
P. tenuifila	T39	-51.4167	-30.2936	KP223385	KP223493
P. tenuifila	T40	-51.4167	-30.2936	KP223386	KP223494
P. tenuifila	T44	-51.1533	-29.5994	KP223387	KP223495
P. tenuifila	T45	-51.245	-29.5281	KP223388	KP223496

*GenBank access numbers
